# G_12/13_ Signaling Pathways Substitute for Integrin αIIbβ3-Signaling for Thromboxane Generation in Platelets

**DOI:** 10.1371/journal.pone.0016586

**Published:** 2011-02-10

**Authors:** Kamala Bhavaraju, Parth R. Lakhani, Robert T. Dorsam, Jianguo Jin, Ian S. Hitchcock, Archana Sanjay, Satya P. Kunapuli

**Affiliations:** 1 Department of Physiology, Temple University School of Medicine, Philadelphia, Pennsylvania, United States of America; 2 Department of Pharmacology, Temple University School of Medicine, Philadelphia, Pennsylvania, United States of America; 3 Sol Sherry Thrombosis Research Center, Temple University, Philadelphia, Pennsylvania, United States of America; 4 Department of Medicine, University of California San Diego, La Jolla, California, United States of America; 5 Department of Anatomy and Cell Biology, Temple University School of Medicine, Philadelphia, Pennsylvania, United States of America; German Heart Center, Germany

## Abstract

**Background:**

We have previously shown that ADP-induced TXA_2_ generation requires signaling from αIIbβ3 integrin in platelets. Here we observed that, unlike ADP, protease-activated receptor (PAR)-mediated TXA_2_ generation occurs independently of αIIbβ3. PAR agonists, but not ADP, activate G_12/13_ signaling pathways. Hence, we evaluated the role of these pathways in TXA_2_ generation.

**Principal Findings:**

Inhibition of ADP-induced thromboxane generation by fibrinogen receptor antagonist SC57101 was rescued by co-stimulation of G_12/13_ pathways with YFLLRNP. This observation suggested an existence of a common signaling effector downstream of integrins and G_12/13_ pathways. Hence, we evaluated role of three potential tyrosine kinases; c-Src, Syk and FAK (Focal Adhesion Kinase) that are known to be activated by integrins. c-Src and Syk kinase did not play a role in ADP-induced functional responses in platelets. Selective activation of G_12/13_ pathways resulted in the activation of FAK, in the absence of integrin signaling. Interestingly, αIIbβ3-mediated FAK activation occurred in a Src family kinase (SFK)-independent manner whereas G_12/13_ pathway caused FAK activation in a SFK and RhoA-dependent manner. A FAK selective inhibitor TAE-226, blocked TXA_2_ generation. However, in comparison to WT mice, Pf4-Cre/Fak-Floxed mice did not show any difference in platelet TXA_2_ generation.

**Conclusions:**

Therefore, we conclude that differential activation of FAK occurs downstream of Integrins and G_12/13_ pathways. However, the common effector molecule, possibly a tyrosine kinase downstream of integrins and G_12/13_ pathways contributing to TXA_2_ generation in platelets remains elusive.

## Introduction

Platelet activation is an essential component of hemostasis and thrombosis, and involves engagement of complex signaling machinery. Injury to sub-endothelium results in platelet adhesion, and subsequent spreading on exposed collagen. Platelet activation also leads to reorganization of platelet cytoskeleton, release of granular contents from dense and alpha granules and finally culminates in integrin activation leading to platelet aggregation [1], [2], [3]. ADP released from the dense granules and the thromboxaneA_2_ (TXA_2_) generated from activated platelets further act as positive feedback mediators and amplify the initial platelet responses and stabilize the hemostatic plug [4], [5].

TXA_2_ is generated from its precursor arachidonic acid through cycloxygenase pathway [6]. TXA_2_ acts on the TP (Prostanoid) receptors and recruits more platelets to the site of injury. Platelets can be activated by broad range of agonists, which can be further classified as strong or weak. ADP, serotonin, and epinephrine, are considered weak agonists [7] whereas thrombin, SFLLRNP (PAR1 agonist), AYPGKF (PAR4 agonist) [8], and Convulxin (GP VI agonist) [9] are strong agonists. Not only these agonists activate platelets with varied potencies but they also induce distinct signaling pathways. Previous studies have shown that ADP-induced TXA_2_ generation in platelets is integrin-dependent [10] however; it is not known whether stronger agonists such as thrombin depend on integrin-mediated signaling for TXA_2_ generation.

ADP activates two G-protein coupled receptors P2Y_1_ and P2Y_12_, activating G_q_ and G_i_ pathways, respectively [1], [3]. In platelets, neither of the ADP receptors can couple to G_12/13_ proteins whereas PAR receptors couple to G_12/13_ proteins [11]. G_12/13_ pathways have been shown to activate Rho kinase [12] and Src family kinases (SFKs) [13]. Earlier studies have shown that G_12/13_ pathways mediate calcium-independent shape change [12], [14], and play a potentiating role in Akt phosphorylation [13] and dense granule secretion [15]. Co-stimulation of platelets with G_12/13_ and G_i_ also leads to platelet aggregation [16].

Agonist binding to platelet receptors results in complex intracellular signaling events termed as inside-out signaling, which leads to the activation of integrins αIIbβ3 and α2β1. Activated integrins change conformation and bind multivalent ligands such as fibrinogen and von Wilebrand Factor (vWF) [17]. Signaling events from ligand binding to fibrinogen receptor are termed as outside-in signaling which, in turn regulate platelet adhesion, spreading, and clot retraction [18]. Outside-in signaling also causes phosphorylation of β_3_ cytoplasmic tails [19], [20], and activation of Phospholipase C (PLC γ) [21], tyrosine kinases such as c-Src, Syk [21] and Focal adhesion kinase (FAK) [22]. FAK is a 125 kDa protein, expressed in both megakaryocytes and platelets [23]. FAK is tyrosine phosphorylated on six tyrosine residues viz., Y397, Y407, Y576/577, Y861 and Y925 [24].

Other signaling molecules, involved in outside-in signaling are Protein Tyrosine Phosphatase-1B (PTP-1B) [25], Protein Phosphatase 1C (PP1c) [26], Calcium and integrin binding protein (CIB) [27] and Protein Kinase C-β (PKC-β) [28], [29].

In this study we show that, G_12/13_ pathways, cause TXA_2_ generation even when signaling from integrin is blocked. We present an evidence that c-Src and Syk kinase do not play a role in ADP-induced functional responses and FAK can be activated downstream of integrin αIIbβ3 as well as G_12/13_ pathways. However, G_12/13_-mediated activation of FAK occurs via Rho kinase and SFKs, whereas integrin-mediated FAK activation is independent of SFKs. Studies to evaluate the role of FAK in thromboxane generation showed that FAK does not contribute to thromboxane generation downstream of integrins. In conclusion, neither c-Src, Syk nor FAK contribute to integrin-mediated thromboxane generation in platelets. Therefore, the common signaling molecule downstream of integrins and G_12/13_ remains yet to be identified.

## Results

### Regulation of thromboxane generation in platelets by G_12/13_ pathways

**Figure 1:**
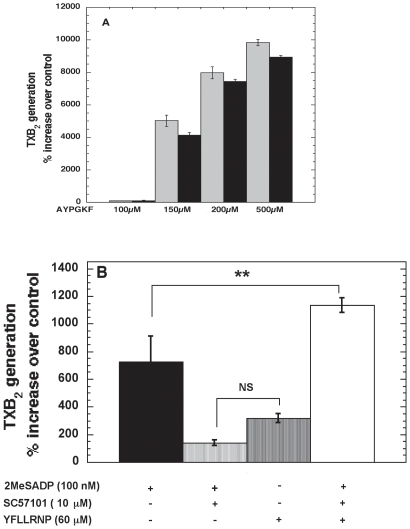
Regulation of thromboxane generation by G_12/13_ pathways. Non-aspirin-treated, washed human platelets were stimulated with different concentrations of AYPGKF in presence and absence of SC57101 (10 µM) (A) or other agonists (B) as indicated for 3.5 minutes at 37°C under stirring conditions (900 rpm) in an aggregometer. After 3.5 minutes the reaction was stopped by snap- freezing in dry ice-methanol bath. TXB_2_ levels were analyzed as described in “Materials and Methods”. The values are representative of 3 independent experiments mean ± S.E.M (n = 3). The data were analyzed by student t-test and ANOVA, ** P≤0.005 was considered significant.

ADP-induced TXA_2_ generation is dependent on integrin activation and as pre-treatment of platelets with fibrinogen receptor antagonist (SC57101) abrogated ADP-induced TXA_2_ generation [10]. We investigated the effect of a fibrinogen receptor antagonist (SC57101) on PAR-mediated TXA_2_ generation. Searle Research developed SC57101 (Skokie, IL) based on the RGDS structure. SC57101 and its analogs are fibrinogen receptor antagonists and do not affect inside out signaling [10]. As shown in Fig. 1A, varying concentrations of AYPGKF, a PAR4 agonist, caused TXA_2_ generation in the presence or absence of SC57101, indicating that PAR agonists cause thromboxane generation independently of integrin signaling. One of the main signaling differences between ADP and PAR agonists is that only PAR agonists can activate G_12/13_ pathways [11]. In order to evaluate the role of G_12/13_ pathways in platelet TXA_2_ generation we co-stimulated platelets with 2MeSADP and/or YFLLRNP. 2MeSADP is a more potent agonist than ADP at the P2Y1 and P2Y12 receptors [30] and YFLLRNP is a weak agonist of PAR1 that selectively activates G_12/13_ pathways (at low doses) [16], [31]. As shown in Fig. 1B, 2MeSADP-induced thromboxane generation was completely blocked in the presence of SC57101. YFLLRNP alone did not cause any significant TXA_2_ generation in human platelets. However, co-stimulation of platelets with 2MeSADP and YFLLRNP in presence of SC57101 caused TXA_2_ generation, indicating that the G_12/13_ pathways can rescue the inhibition rendered by fibrinogen receptor antagonist. Hence, G_12/13_ pathways can substitute for integrin-mediated signaling by probably activating similar effector molecules.

### Role of c-Src and Syk downstream of integrin αIIbβ3 in thromboxane generation in platelets

**Figure 2:**
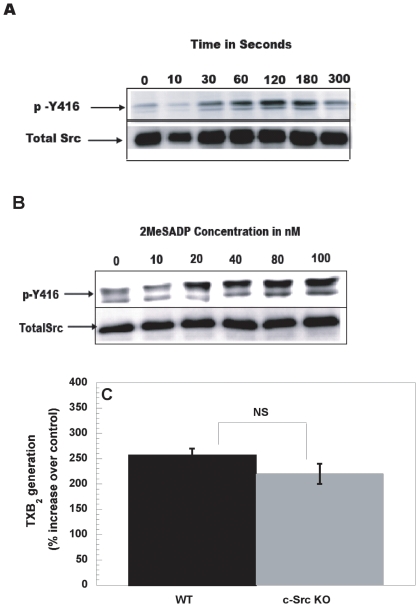
Role of c-Src and Syk downstream of integrin αIIbβ3 in thromboxane generation in platelets. Aspirin–treated, washed platelets were stimulated with 2MeSADP (100 nM) at various time points (A) or with varying concentrations of 2MeSADP (B) for one minute under stirring conditions at 37°C. The lysates were then subjected to western blotting analysis and probed with anti-phospho-(Y416) and total c-Src antibodies as lane loading control. Washed murine (WT or c-Src KO) platelets, without aspirin-treatment, were stimulated with 2MeSADP (100 nM) for 3.5 minutes and TXB_2_ levels were analyzed (C) as described for Figure 1. The data are represented as the % Fold increase over the control. Aspirin-treated washed platelets were stimulated with 2MeSADP (100 nM) for (30–120 seconds) or convulxin (100 ng/ml) for 30 seconds under stirring conditions at 37°C (D). The lysates were then subjected to western blotting analysis and probed with anti- phospho- Syk(Y 525/526) and total Syk antibodies as lane loading control. The data are representative of at least 3 separate experiments.

Previous studies have shown that c-Src and Syk are signaling effectors in platelets that are regulated by integrins [32]. In order to identify the common signaling molecules downstream of fibrinogen receptor and G_12/13_ pathways, we evaluated the role of these individual kinases in ADP-induced TXA_2_ generation. We reasoned that as these tyrosine kinases are activated downstream of fibrinogen receptor and because ADP-induced thromboxane generation requires signaling events from fibrinogen receptor, at least one of these kinases could be crucial for ADP-induced thromboxane generation. 2MeSADP-induced phosphorylation of c-Src Y416 occurred in a time and concentration-dependent manner (Figs. 2A and B). However, 2MeSADP-induced TXA_2_ generation in wild type mice was not different from the c-Src knockout mice (Fig. 2C), suggesting that either other Src family members may compensate for the absence of c-Src or that c-Src activation downstream of ADP receptors is not involved in TXA_2_ generation. We then studied Syk kinase activation downstream of ADP receptors. Syk kinase was not activated downstream of ADP receptors (Fig. 2D), as determined by the phosphorylation of the Tyr525/526 residues [33]. Taken together, these results suggest, that c-Src and Syk may not be not essential for ADP-induced thromboxane generation in platelets.

### Focal Adhesion Kinase is activated downstream of integrins and G_12/13_ pathways

**Figure 3:**
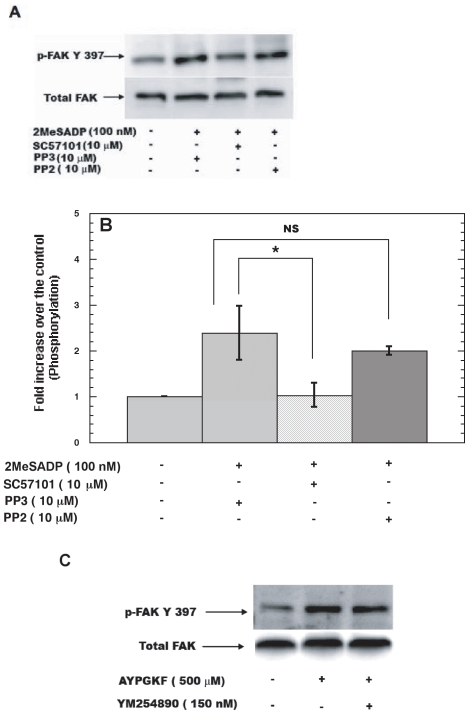
Focal Adhesion Kinase is activated downstream of integrins and G_12/13_ pathways. Aspirin treated, washed platelets were stimulated with 2MeSADP (100 nM) in presence or absence of reagents (as indicated) for 60 seconds under stirring conditions at 37°C (A). The lysates were then subjected to western blotting analysis and probed with anti- phospho- FAK (Y-397) and total FAK antibodies as lane loading control. The data are representative of mean ± S.E.M (n = 3). The data was analyzed by ANOVA and * P≤0.05 was considered significant (B). Aspirin-treated washed platelets were stimulated with AYPGKF (500 µM) in presence or absence of YM254890 (150 nM) (C), The lysates were then subjected to western blotting analysis and probed with anti- phospho- FAK (Y-397) and total FAK antibodies as lane loading control.

We next evaluated the activation of FAK by ADP receptors, using Y397 phosphorylation as an activation marker. FAK contains multiple tyrosine phosphorylation sites and the sequential tyrosine phosphorylations of these sites causes complete FAK activation beginning with autophosphorylation Y397 phosphorylation [34]. As shown in Figs. 3A & B, FAK is activated downstream of ADP receptors and this activation is blocked by a fibrinogen receptor antagonist but not by a pan SFK inhibitor PP2. These results indicate FAK activation by ADP occurs in an integrin-clustering-dependent manner, independent of SFKs.

We further investigated FAK activation downstream of G_12/13_ pathways. PAR agonists activate both G_q_ and G_12/13_ pathways in platelets [11]. The G_q_ pathways can be inhibited by YM254890 [35] without affecting the G_i_ or G_12/13_ pathways [13]. Since YFLLRNP is a weak, agonist for G_12/13_ pathways at low concentrations and at higher concentrations can also activate G_q_ pathways [16], we chose to stimulate platelets with AYPGKF (500 µM) in the presence of YM254890 as tool for selective and strong activation of G_12/13_ pathways for our phosphorylation studies [13]. As shown in Fig. 3C, AYPGKF caused activation of FAK in human platelets as determined by the phosphorylation of Y397 residue. This phosphorylation was unaffected in the presence of YM254890. It is important to note that 150 nM of YM254890 abrogates AYPGKF-induced platelet aggregation and secretion [13] and hence autophosphorylation of FAK on Y397 is through G_12/13_ pathways but not by outside-in signaling.

### Signaling pathways regulating FAK activation downstream of G_12/13_ pathways

**Figure 4:**
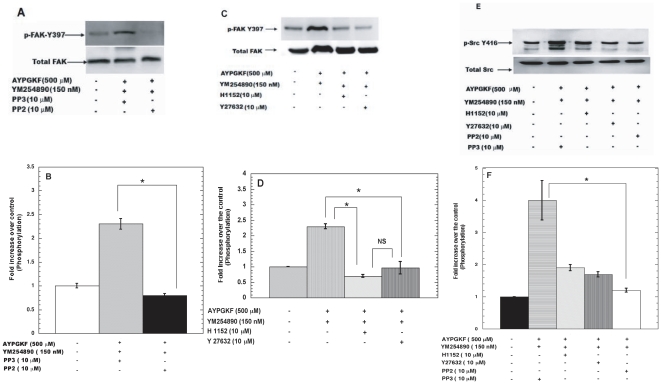
Signaling pathways regulating FAK activation downstream of G_12/13_ pathways. Aspirin-treated, washed human platelets were pre-treated with different inhibitors (as indicated) for 5 minutes at 37°C followed by stimulation with AYPGKF (500 µM) for 90 seconds under stirring conditions at 37°C in an aggregometer. The lysates were then subjected to western blotting analysis and probed with anti- phospho FAK (Y-397) and total FAK antibodies as lane loading control (A &C) or anti- phospho- Src (416) and total c-Src antibodies as lane loading control (E). Quantitative data, normalized to the lane loading control, are representative of mean ± S.E.M (n = 3). The data was analyzed by ANOVA and * P≤0.05 was considered significant (B, D, and F).

We have shown that G_12/13_ pathways activated by AYPGKF in the presence of YM254890 can activate FAK (Fig. 3A). Under these conditions we used pharmacological inhibitors to evaluate the signaling molecules that could regulate FAK phosphorylation. As shown in Figs. 4A&B, PP2, a pan SFK inhibitor abolished FAK phosphorylation mediated by G_12/13_ pathways. Our results from Figs. 4C&D show that Rho kinase inhibitors H1152 and Y27632 also markedly inhibited G_12/13_-mediated FAK phosphorylation. These results indicate that both SFKs and Rho kinase play an important role in FAK activation downstream of G_12/13_ pathways in platelets. In order to further delineate the signaling pathways downstream of G_12/13_, we also studied Src Y416 phosphorylation in presence of Rho kinase inhibitors. As shown in Figs. 4E&F, G_12/13_-mediated Src Y416 phosphorylation was dramatically inhibited in presence of Rho kinase inhibitors H1152 and Y27632, thus suggesting that SFKs are activated downstream of Rho kinase in the G_12/13_ signaling cascade.

### Evaluation of FAK as a common signaling effector molecule regulating thromboxane generation downstream of integrins and G_12/13_ pathways

**Figure 5:**
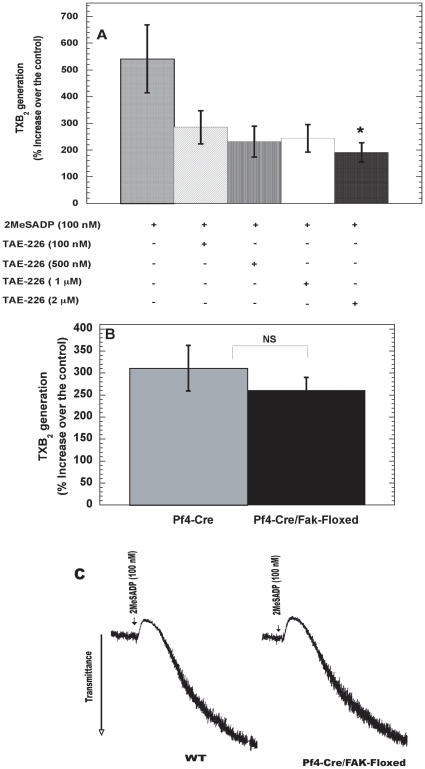
Evaluation of FAK as a common signaling effector molecule regulating thromboxane generation downstream of integrins and G_12/13_ pathways. Non-aspirin-treated, washed human platelets were pre-treated with varying concentrations of TAE-226 for 5 minutes at 37°C (A) and murine platelets from WT and Pf4-Cre/Fak-Floxed mice (B) were stimulated with 2MeSADP (100 nM) for 3.5 minutes and TXB_2_ levels were analyzed as described for Figure 1. Aggregation tracings were measured from WT and Pf4-Cre/Fak-Floxed mice and representative tracings are shown (C). The values are representative of 3 independent experiments mean ± S.E.M (n = 3). The data were analyzed by ANOVA and student t-test, * P≤0.05 was considered significant.

We next evaluated whether activated FAK played a role in ADP-induced TXA_2_ generation. TAE-226 has recently been identified as a selective inhibitor of FAK with an IC_50_ of 5.5 nM [36]. To determine the effect of FAK inhibition on ADP-induced thromboxane generation platelets were treated with varying concentrations of TAE-226. As demonstrated in Fig. 5A, TXA_2_ generation was significantly inhibited by TAE-226 at a higher concentration of 2 µM. Pharmacological inhibitors are often known to have off target and broad-spectrum effects. The specificity of TAE-226 was never evaluated in platelets and reports suggest that TAE-226 inhibits Pyk2 (a Focal Adhesion kinase family member) with an IC_50_ of 5 nM [37]. Hence, we studied thromboxane generation in WT and Pf4-Cre/FAK-floxed mice platelets. Murine platelets from WT and Pf4-Cre/FAK-floxed were stimulated with 100 nM of 2MeSADP and thromboxane levels were measured from WT and Pf4-Cre/FAK-floxed mice samples. As shown in Fig. 5B there was no significant difference observed thromboxane levels and aggregation tracings (Fig. 5C) between WT and Pf4-Cre/FAK-floxed. These results suggest that TAE-226 might exhibit some non-specific effects, and FAK is not the common signaling molecule regulating thromboxane generation downstream of integrins and G_12/13_ pathways.

## Discussion

Activated platelets release positive feedback mediators such as ADP and TXA_2_. The molecular mechanisms involved in thromboxane generation by different platelet agonists are not clearly understood. Weaker agonists such as ADP require outside-in signaling through activated fibrinogen receptor to cause thromboxane generation in platelets. Patients with Glanzmann's thrombasthenia show defective thromboxane generation with ADP but not with thrombin [38]. Our results show that PAR agonists mediate TXA_2_ generation independent of the integrin signaling. Previous studies by Stefanini *et al* [39] showed that stimulation of platelets with high and low doses of GPVI agonist convulxin in the presence of integrin blockers showed a marginal decrease in thromboxane generation, indicating that thromboxane generation downstream of GPVI receptors is not solely dependent on integrins. Thus, these results indicate that platelets can generate thromboxane in an integrin-dependent and-independent manner. Since PAR receptors can activate G_12/13_ pathways we reasoned that there might be a common signaling molecule, which could be activated by both G_12/13_ pathways (which are not activated by ADP) and integrins. This common signaling molecule might regulate TXA_2_ generation downstream of G_12/13_ pathways independent of integrins.

It has been known that several platelet agonists cause activation of G_12/13_, but not much is known about the intracellular signaling pathways downstream of this activation. Studies from our group have shown that G_12/13_ pathways activate Src family kinases in platelets [13]. Klages *et al* [12] showed that G_12/13_ proteins can activate tyrosine kinases such as Syk and c-Src. Interestingly these kinases are also known to be activated downstream of outside-in signaling [32]. Although integrins and G_12/13_ pathways activate c-Src, it is clear that this kinase has no significant role in thromboxane generation downstream of ADP receptors (Fig. 2C). Interestingly, thromboxane A_2_, an agonist that couples to G_q_ and G_12/13_ [11], still caused the phosphorylation of p72Syk in G_q_-deficient mice [12]. This result suggests that activation of Syk might occur downstream of G_12/13_ signaling. However, Syk does not appear to be activated downstream of ADP receptors (Fig. 2D). Hence, both c-Src and Syk can be ruled out as the common signaling molecule mediating thromboxane generation downstream of G_12/13_ and integrin pathways.

**Figure 6:**
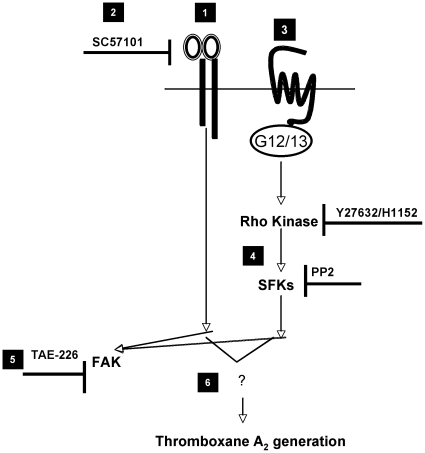
Model depicting the regulation of TXA_2_ generation by G_12/13_ pathways and integrins through FAK. Integrin clustering leads to FAK activation (1). Fibrinogen receptor antagonist SC57101 prevents integrin clustering hence inhibits FAK activation (2). FAK can be activated in an integrin-independent manner by G_12/13_ pathways (3). FAK is activated downstream of Rho kinase and SFKs upon stimulation of G_12/13_ pathways (4). FAK can be inhibited by TAE-226 (5). Common effector molecule downstream of integrins and G_12/13_ pathways contributing to thromboxane generation are unknown (6).

Our studies show that FAK is differentially activated downstream integrins (Figs. 3A and B) and G_12/13_ pathways (Figs. 3C, 4A and B). However, recently it was reported by Gong *et al* [40] that G_13_ binds to integrin αIIbβ3 and mediates outside-in signaling. Gong *et al* claim that Rho activation downstream of PARs and integrins is temporally and spatially regulated and mediates opposing effects. With regards to FAK activation in platelets it does not seem to be the probable mechanism since ADP-induced FAK activation is independent of SFKs (Figs. 3A and B) and downstream of G_12/13_ pathways FAK activation is SFK and Rho-dependent (Figs. 4A and 4C). Thus, there maybe some other integrin-mediated signaling pathways regulating FAK activation in platelets. Upstream of FAK, SFKs are activated by G_12/13_ pathways [13]. SFKs regulate FAK activation downstream of G_12/13_ pathways but not downstream of integrin signaling (Figs. 4A and 3A). Although integrin signaling leads to c-Src and FAK activation, our results suggest that Src/Syk pathways and FAK pathways are independently activated by integrins as outlined in Fig. 6, with possible differential functional implications in platelets.

G_12/13_ pathways are known to activate RhoA/Rho kinase pathways leading to calcium-independent shape change [12], [14]. These pathways are also known to regulate dense granule release through RhoA/Rho kinase pathways [15]. Our studies show that Rho kinase pathways regulate FAK activation through SFKs (Figs. 4E&F). This is a novel observation and indicates that Rho kinase pathways are the nodal point of SFK activation, ppIMδ phosphatase, and other pathways regulating dense granule release. We postulate that these pathways are independent of each other as; inhibition of SFKs (with PP2) has no significant effect on PAR-mediated shape change or dense granule release in aspirin-treated platelets [41]. Hence, SFK activation occurs downstream, rather than upstream, of RhoA/Rho kinase pathways upon G_12/13_ stimulation as outlined in Fig. 6. If SFK activation were to occur upstream of Rho kinase pathways, then SFK inhibition would have affected platelet shape change as well as dense granule release reaction in aspirin-treated platelets.

Furthermore, complementary approaches e.g. pharmacological FAK inhibitor TAE-226 and Pf4-Cre/Fak-Floxed mice were employed to evaluate role of FAK in thromboxane generation. Pre-treatment of platelets with various doses of TAE-226 did not lead to significant decrease in thromboxane levels. However, inhibition of thromboxane generation was observed at 2 µM concentration only (Fig. 5A). Similarly thromboxane generation was not affected in Pf4-Cre/Fak-Floxed mice, when compared to WT littermates (Fig. 5B). These results indicate that FAK does not contribute to thromboxane generation downstream of integrins and TAE-226 might have some non-specific effects on platelets.

Since our data indicates that loss of FAK does not translate into diminished thromboxane generation, it is possible that some other tyrosine kinases might be compensating for the loss of FAK in platelets, however it might be unlikely since previous studies in Pf4-Cre/Fak-Floxed mice showed differences in tail bleeding times and platelet spreading and no upregulation of Pyk2 expression was observed in FAK-Floxed mice [23]. Recently, another FAK inhibitor PF-573,228 was shown to inhibit platelet aggregation [42]. However, our platelet aggregation studies comparing WT and Pf4-Cre/FAK-Floxed mice did not show any differences in aggregation (Fig. 5C). Furthermore, as our previous studies have shown that ERK can be activated even in the presence of integrin antagonists [43] therefore, ERK cannot be the common effector downstream of integrins and G_12/13_ pathways.

Thus, we conclude that G_12/13_ pathways through a Rho kinase/SFK dependent manner activate FAK. However, FAK activation downstream of integrins occurs independently of SFKs. Finally none of the three-tyrosine kinases c-Src, Syk or FAK seems to play a role in thromboxane generation downstream of integrins. Thus, the common signaling effector, possibly a tyrosine kinase, contributing to thromboxane downstream of G_12/13_ pathways and integrins remains yet to be identified.

### Ethical Statement

Approval for this study was obtained from the Institutional Review Board of Temple University (Philadelphia, PA), and mice were used for physiological measurements using the protocol ID number 3364, approved by the Institutional Animal Care and Use Committee (IACUC).

## Materials and Methods

### Materials

2MeSADP, Apyrase grade VII, human fibrinogen, acetylsalicylic acid, were obtained from Sigma (St. Louis, MO). SC57101 was gift from Searle Research and Development (Skokie, IL). Hexapeptide AYPGKF was custom synthesized at Invitrogen (Carlsbad, CA). Convulxin was purchased from Centerchem Inc. (Norwalk, CT). The heptapeptide, YFLLRNP was synthesized by New England Biolabs (Beverly, MA) or by Research Genetics (Huntsville, AL). Phospho-specific antibodies against Y416 Src family, anti-Syk Y525/526, Total Src and Total Syk were obtained from Cell Signaling Technologies (Beverly, MA). Antibodies against FAK Y397 were obtained from Millipore, (Bedford, MA) and Total FAK was from Biosource (Camarillo, USA). PP2 and PP3 were purchased from Biomol (Plymouth Meeting, PA). The Rho kinase inhibitor H1152 was obtained from Toronto Research Chemicals (North York, ON). Y-27632 was obtained from Calbiochem (San Diego, CA). YM-254890 was a gift from Yamanouchi Pharmaceuticals Co., Ltd (Ibaraki, Japan). TAE-226 was generously provided by (Novartis Pharma AG, Switzerland). The other reagents were of reagent grade, and de-ionized water was used throughout.

### Animals

8–12 weeks old Pf4-Cre, and Pf4-Cre/FAK-floxed mice were generated in accordance with previously described protocol [44]. The generation of Src KO mice was described previously [45] and these mice along with wild type littermates in C57BL/6 background were used in the experiments.

### Preparation of washed human and murine platelets

All experiments with human volunteers were performed in accordance with Declaration of Helsinki. Whole blood was drawn from healthy, human volunteers selected from students, staff or workers at the Temple University with written informed consent. Donated blood was collected in tubes containing one-sixth volume of acid citrate dextrose (ACD) (2.5 g of sodium citrate, 1.5 g of citric acid, and 2 g of glucose in 100 ml of de-ionized water). Citrated blood was centrifuged and platelets were isolated with previously established protocol [46].

### Isolation of mouse platelets

Blood was collected from the vena cava of anaesthetized mice into syringes containing one-tenth blood volume of 3.8% sodium citrate as anticoagulant. Red blood cells were removed by centrifugation at 100× *g* for 10 min. PRP was recovered, and platelets were pelleted at 400× *g* for 10 min. The platelet pellet was resuspended in Tyrode's buffer (pH 7.4) containing 0.01 unit/ml apyrase. The isolated platelets were subsequently used for experiments.

### Western blot analysis

Aliquots of aspirin-treated, washed human platelets were lysed using Laemmli buffer in presence of dithiothreitol (DTT) (100 mM) and boiled for 10 min. The platelet lysates were loaded onto a 10% -Tris-glycine gel, subjected to SDS-PAGE (Sodium Dodecyl Sulfate- Polyacrylamide Gel Electrophoresis), and transferred to PVDF membrane. Nonspecific binding sites were blocked by incubating the membrane in Tris–buffered saline-Tween (TBST; 20 mM Tris, 140 mM NaCl, 0.1% (vol/vol) Tween 20) containing 3% (wt/vol) bovine serum albumin (BSA) and 5% (vol/vol) Irish cream for 30 min at RT, followed by incubating it overnight at 4°C with gentle agitation in the primary antibody (1∶1000 dilution for anti-FAK Y397, anti-Src Y416, anti-Syk Y525/526, anti-Syk, Anti-FAK and anti-Src in TBST with 3% BSA). After washing with TBST, the membranes were probed with an alkaline phosphatase-labeled secondary antibody (1∶5000 dilutions in TBST with 3% BSA) for 1 hour at RT. After additional washing steps, membranes were incubated with chlordiazepoxide (CDP)–Star® chemiluminescent substrate (Tropix, Bedford, MA) for 10 min at RT, and immunoreactivity was detected using a Fuji Film Luminescent Image Analyzer (LAS-3000 CH; Tokyo, Japan).

### Measurement of TXA_2_ generation in human and mice platelets

Washed human platelets (500 µl brought to a concentration of 2×10^8^ platelets/mL) were stimulated with 2MeSADP (100 nM) in a lumi-aggregometer at 37°C with stirring at 900 rpm, in the presence or absence of TAE-226. Similarly murine platelets from WT and Pf4-Cre/FAK-floxed mice (250 µl brought to a concentration of 2×10^8^ platelets/mL) were stimulated with 2MeSADP (100 nM). After 3.5 min of stimulation, the reaction was stopped by quickly freezing the sample in a dry ice-methanol bath. The level of TXB_2_, the stable metabolite of TXA_2_ was measured using a previously established protocol [47].

### Statistical Analysis

The results were quantified, expressed as mean ± S.E.M. The data was statistically analyzed using Student's t-test and ANOVA. *P≤0.05/ ** P≤0.05 were considered significant.
